# Endoplasmic reticulum stress: bridging inflammation and obesity-associated adipose tissue

**DOI:** 10.3389/fimmu.2024.1381227

**Published:** 2024-04-04

**Authors:** Kaile Ma, Yanjiao Zhang, Jingyi Zhao, Lijuan Zhou, Min Li

**Affiliations:** Institute of Metabolic Diseases, Guang’anmen Hospital, China Academy of Chinese Medical Sciences, Beijing, China

**Keywords:** endoplasmic reticulum stress, adipose tissue, obesity, inflammation, metabolic disorder

## Abstract

Obesity presents a significant global health challenge, increasing the susceptibility to chronic conditions such as diabetes, cardiovascular disease, and hypertension. Within the context of obesity, lipid metabolism, adipose tissue formation, and inflammation are intricately linked to endoplasmic reticulum stress (ERS). ERS modulates metabolism, insulin signaling, inflammation, as well as cell proliferation and death through the unfolded protein response (UPR) pathway. Serving as a crucial nexus, ERS bridges the functionality of adipose tissue and the inflammatory response. In this review, we comprehensively elucidate the mechanisms by which ERS impacts adipose tissue function and inflammation in obesity, aiming to offer insights into targeting ERS for ameliorating metabolic dysregulation in obesity-associated chronic diseases such as hyperlipidemia, hypertension, fatty liver, and type 2 diabetes.

## 1 Introduction

Obesity is a multifactorial chronic disease that prevails worldwide. According to the World Health Organization (WHO), it is estimated that by 2025, one-fifth of the global adult population will be obese (1), approximately 39% of adults will be categorized as overweight, and around 13% of adults will be classified as obese (2). Obesity stands out as a pivotal risk factor for a multitude of chronic metabolic conditions, including diabetes, insulin resistance, fatty liver disease, hyperlipidemia, chronic kidney disease, cardiovascular disease, hypertension and inflammation (3). It significantly escalates the incidence of common ailments like cardiovascular diseases and type 2 diabetes. The dangers of obesity are self-explanatory (4). Endoplasmic reticulum stress (ERS) is considered a potential target for the treatment of inflammation, obesity and metabolic disorders (5).

The endoplasmic reticulum (ER) is an organelle located in eukaryotic cells. It plays a crucial role in protein synthesis, folding, maturation, and the proper transport of correctly folded proteins (6). Additionally, the ER interacts with the Golgi apparatus, mitochondria, lysosomes, phagosomes, and the cell nucleus (7). ERS is characterized by the excessive accumulation of unfolded proteins or misfolded proteins, triggering the unfolded protein response (UPR), which involves the activation of transcription within the organism. Ultimately, this leads to the restoration of ER homeostasis and the amelioration of ERS-related diseases (8). The UPR signaling pathway consists of three main branches: PERK (protein kinase R-like ER kinase, encoded by the EIF2AK3 gene), IRE1 (inositol-requiring enzyme 1, encoded by the ERN1 gene), and ATF6 (activating transcription factor 6) (9). The initial ER chaperone protein, immunoglobulin heavy chain binding protein/glucose-regulated protein 78 (BiP/GRP78), resides on the ER membrane. Its primary function is to bind to transducers of the three branches of the UPR pathway and maintain them in an inactive state (10). Amid ER, BiP/GRP78 is displaced by interactions with misfolded proteins, instigating the liberation and activation of IRE1, PERK, and ATF6. Ultimately, the three arms of the UPR pathway clear misfolded proteins, hinder protein synthesis, foster ERS biosynthesis, and prompt the transcription of ERS-associated chaperone genes (11). Numerous studies indicate that ERS plays a crucial role in obesity. ER activation is closely associated with obesity and inflammation, exerting a significant impact on the functionality of adipose tissue in obesity (7, 12, 13).

Obesity is considered a chronic inflammatory state characterized by excessive or abnormal accumulation of fat in adipose tissue (14). This is primarily manifested by the infiltration and activation of immune cells in metabolic organs such as adipose tissue (15). The initial signals of obesity-related inflammation is believed to stem from excessive metabolism, namely nutrient and energy surplus. Therefore, the chronic inflammation associated with obesity is also referred to as metabolic inflammation (16). As a detrimental factor, obesity significantly impairs the normal function of the ER, imposing immense pressure on the UPR and inducing misfolding of proteins, ultimately leading to ERS. Simultaneously, ERS can lead to excessive generation of reactive oxygen species and disruption of energy metabolism, ultimately resulting in oxidative stress and inflammation (17). ERS is significantly elevated in both obese individuals and obese mice adipose tissue. This elevation is a key mechanism by which obesity induces the activation of inflammatory and related stress responses such as JNK (c-Jun N-terminal kinase), IKK (Inhibitor of KappaB Kinase), and NF-κB (nuclear factor-kappa B). The different branches of the UPR are sequentially activated under the crosstalk between ERS and inflammation processes, playing significant roles in obesity. This paper provides a comprehensive review of the mechanisms underlying the crosstalk between ERS and obesity-related inflammation, particularly within adipose tissue, with the aim of fully exploring the therapeutic potential of targeting ERS for obesity treatment.

## 2 The interaction between adipose tissue functionality and ERS in obesity

### 2.1 ERS in obesity

**Figure 1:**
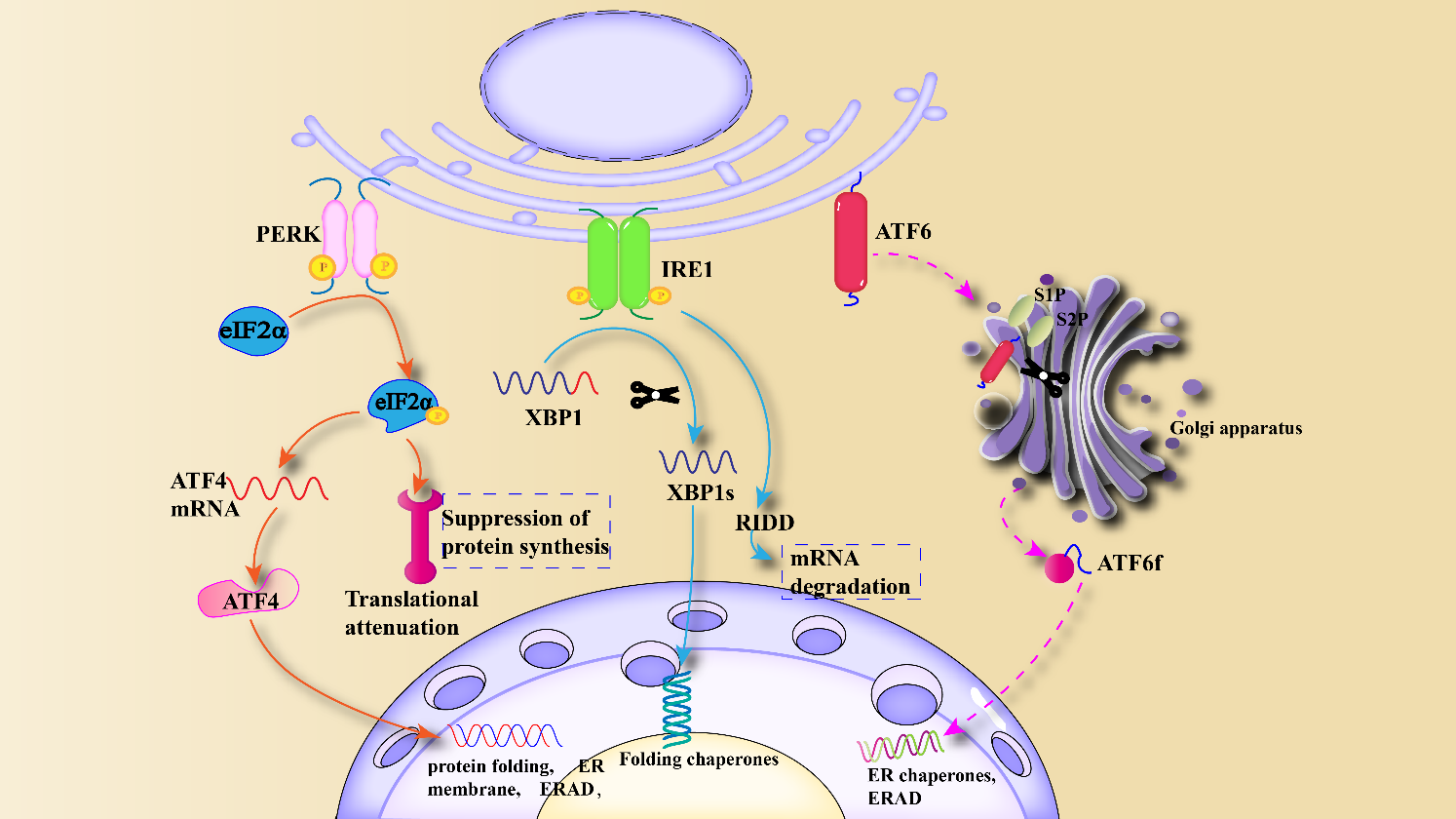
Mechanism of ERS and unfolded protein response: Under ERS, UPR is activated, causing the activation of three downstream branches, PERK, IRE1 and ATF6, and stimulating genes and transcription factors related to protein folding and ERAD.

Obesity is characterized by a state of low-grade chronic inflammation, primarily disrupting normal metabolic functions within the body. It involves not only the regulation of glucose homeostasis and lipid pathways but also the integration of immune responses and metabolic pathways. Dysfunction of the ER is a key characteristic of these metabolic disorders. The ER regulates various cellular processes through the UPR signaling pathway, including nutrient metabolism, cell proliferation and death, inflammation, and insulin signal transduction (18). The UPR is primarily utilized to restore protein homeostasis and is one of the hallmarks of chronic inflammation in adipose tissue of obese individuals. Under ERS, IRE1 oligomerizes and undergoes autophosphorylation, inducing the cytoplasmic endonuclease (RNase) activity (19). After activation, IRE1 induces splicing of XBP1 (X-box binding protein 1) mRNA through its RNase domain, resulting in the excision of a 26-base pair fragment to adapt to ERS (20). XBP1 is a powerful transcription factor that enters the nucleus to bind specific DNA and initiate the expression of genes involved in lipid production and protein folding, crucial for responding to ERS effectively (21). Prolonged ERS can disrupt this pathway, leading to abnormal lipid accumulation and dysfunction of adipocytes, thereby promoting obesity-related diseases. The cytoplasmic domain of PERK is a chaperone protein that can detect ERS and lead to PERK autophosphorylation (22). PERK’s potential therapeutic mechanism against excessive ERS is through reducing the translation of misfolded proteins, thereby decreasing the influx of new proteins into the ER compartment filled with misfolded proteins (23). When PERK is activated, its target protein eIF2α (eukaryotic translation initiation factor 2, alpha subunit) is activated. Overexpression of the target protein eIF2α and its downstream branches in cells can impair protein synthesis through various mechanisms (24). Simultaneously, PERK-mediated eIF2α phosphorylation is associated with abnormal glucose tolerance. Reduced PERK activity promotes insulin secretion in response to glucose stimulation, thereby influencing insulin sensitivity and β-cell function (25). ATF6 is a transmembrane transcription factor with both C-terminal and N-terminal domains. Under ERS, ATF6 is translocated to the Golgi apparatus, where it is cleaved by proteases S1P (Sphingosine-1-phosphate) and S2P (Sphingosine-2-phosphate), releasing its cytoplasmic domain (ATF6f) (19). The N-terminal cytoplasmic fragment of ATF6 can translocate to the nucleus under the guidance of a nuclear localization signal, where it binds to downstream factors of other branches and transfers to the nucleus. Inside the nucleus, it serves as a transcription factor to induce the transcriptional expression of various ERS genes [such as CHOP (C/EBP homologous protein) and GADD (Growth Arrest and DNA Damage)], thereby restoring normal metabolic function of the ER (26). Internal ERS in the obese organism is illustrated in **Figure 1**. Obesity can induce central and peripheral ERS, activating the UPR pathway, where inflammation mechanisms such as JNK, NF-κB play critical roles in metabolic disorders. Therefore, targeting ERS may be a potential therapeutic target for obesity (27). Obesity-induced excessive lipid stimulation triggers ERS. In obesity, inflammatory signals, including those generated by excess lipids, can stimulate ERS and inflammation reactions in multiple cells, playing a crucial role in metabolic disorders (28). The effects of ERS and inflammation pathways in obesity directly or indirectly disrupt the metabolic functions of several tissues, including glucose and lipid metabolism, underscoring the importance of understanding ER homeostasis and the potential therapeutic role of ERS in obesity.

### 2.2 The relationship between adipose tissue function and ERS

#### 2.2.1 Basic function of adipose tissue

Adipose tissue exhibits metabolic activity within the human body and plays a pivotal role in the treatment of metabolic disorders and their complications (29), such as obesity (30). It is known that mammals have three types of adipose tissue. White adipose tissue serves as the primary site for energy storage and release, and it secretes hormones and cytokines that regulate insulin resistance (31). Additionally, it is a major site for mobilizing lipid distribution and metabolism throughout the body (32). The imbalance between long-term nutrient intake and energy expenditure results in an increase in the size (hypertrophy) and number (hyperplasia) of adipocytes, leading to the expansion of white adipose tissue and the development of obesity (33). Brown adipose tissue mediates non-shivering thermogenesis by expressing tissue-specific uncoupling protein 1 (UCP1) in mitochondria (34), resulting in a significant elevation of body temperature above ambient temperature. This process promotes adaptive thermogenesis, ATP (adenosine triphosphate) generation, and substrate oxidation (35). Beige adipose tissue functions intermediate to the other two fat depots, primarily involved in generating heat to maintain body temperature (36). Adipose tissue, serving as a molecular network linking obesity, adipokine secretion, chronic inflammation, insulin resistance, and ERS, can secrete a variety of adipokines, including interleukin-1β (IL-1β), interleukin-6 (IL-6), tumor necrosis factor-α (TNF-α), leptin, adiponectin, Fibroblast Growth Factor 21 (FGF21), and vascular endothelial growth factor(VEGF), among others. These adipokines are closely associated with obesity, diabetes, insulin resistance and inflammation (37).

#### 2.2.2 The interaction between adipose tissue function and ERS

Key features of insulin resistance induced by obesity include alterations in inflammatory signaling in adipocytes (38) and infiltration of immune cells into adipose tissue (39). Adipose tissue can also contribute to insulin resistance by disrupting insulin signaling. The inflammatory cytokines secreted by adipose tissue macrophages and adipocytes can activate inflammatory signaling pathways, such as JNK (40), thereby promoting the degradation of insulin receptor substrate 1 (IRS-1) and the binding of IRS-1 to the insulin receptor (41). Pathological expansion of adipose tissue leads to abnormal hypertrophy and thickening of adipocytes, resulting in adipocyte hypoxia, chronic low-grade inflammation, reduced vascularization, decreased reactive oxygen species production, and ERS (42). ERS, mitochondrial dysfunction, and oxidative stress are all directly associated with adipose tissue dysfunction (43). ERS plays a role in the modification, folding, and transport of proteins. Abnormalities in ER function can significantly impair the physiological function of adipose tissue, which serves as a vital endocrine organ (44).

##### 2.2.2.1 ERS regulates lipogenesis

**Figure 2:**
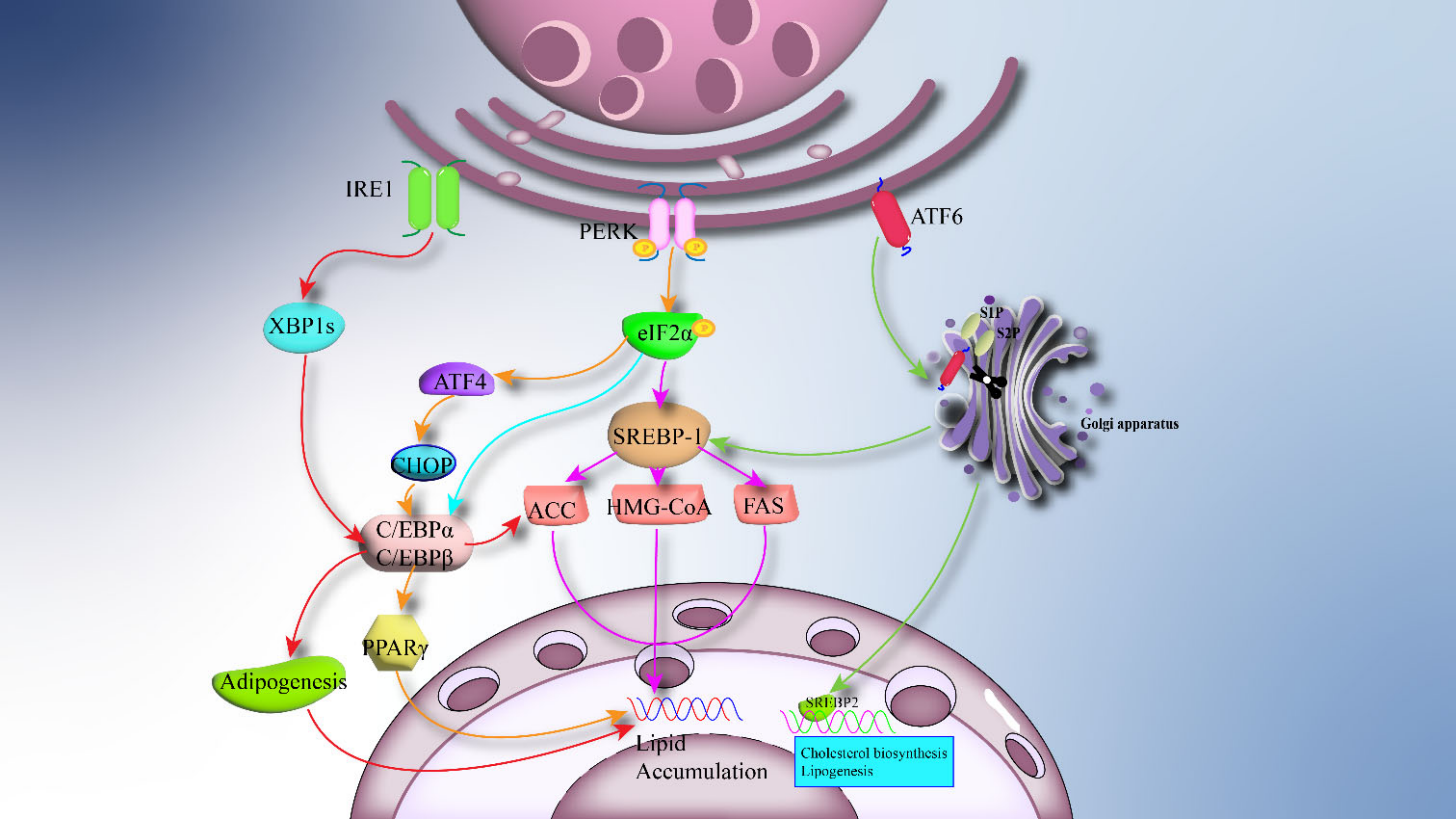
ERS regulates adipogenesis: The overactivation of ERS in adipose tissue causes downstream UPR to regulate adipogenesis. IRE1 stimulates CCAAT/enhancer binding protein-A (C/EBPa) to promote lipogenesis and related transcription factors via XBP1s. PERK and ATF6 regulate lipogenesis by 1/Sterol regulatory element binding protein 1 (SREBP-1).

ERS can regulate the formation and differentiation of adipocytes through different branches, including the synthesis of triglycerides, fatty acids, and cholesterol. This process is mainly regulated by transcription factors, sterol regulatory element-binding proteins (SREBPs), and acyltransferase (DGATs) (45). The PERK and IRE1 arms of the UPR play a crucial role in adipogenesis and differentiation, while the ATF6 arm is closely associated with the process of adipogenesis (46). Research has indicated that the downstream target factor XBP1 of the IRE1 branch is highly expressed in adipocytes (47), and it can control the activity and expression of key enzymes involved in phospholipid biosynthesis (48). Transcription factor CCAAT-enhancer-binding protein alpha (C/EBPα) and transcription factor CCAAT-enhancer-binding protein beta (C/EBPβ) are critical regulatory factors in adipogenesis, and the IRE1/XBP1 pathway regulates adipogenesis through interaction with the C/EBP family transcription factors (49). During early adipogenesis, C/EBP-β induces the expression of mRNA encoding the inactive form of the transcription factor XBP-1 (XBP-1u). Upon activation of the UPR, XBP-1u mRNA undergoes unconventional splicing by IRE1 to generate mRNA encoding the active XBP-1 protein (XBP-1s). In turn, XBP-1s binds and activates the promoter of key adipogenic transcription factor C/EBP-α, playing a crucial role in adipogenesis (46). The IRE1/XBP1 branch has been shown to be activated in mice fed a high-sugar diet, directly influencing the expression of genes related to fatty acid production, such as stearoyl-CoA desaturase 1 (SCD1), diacylglycerol acyltransferase 2 (DGAT2), and acetyl-CoA carboxylase 2 (ACC2). Mice lacking XBP1 in the liver exhibit severe hypotriglyceridemia and hypocholesterolemia due to reduced lipogenesis, further demonstrating the importance of the IRE1/XBP1 branch in fatty acid synthesis (50). The PERK branch is extensively studied, and it regulates adipogenesis through downstream phosphorylation of eIF2α. Both *in vivo* and *in vitro* studies have demonstrated that phosphorylation of eIF2α, in response to ERS, can inhibit the development of adipocytes (51). Activating transcription factor 4 (ATF4) and sterol regulatory element-binding protein 1 (SREBP-1) are downstream branches of eIF2α, primarily responsible for regulating the biosynthesis of fatty acids and triglycerides (52). SREBP-1 can induce the transcription of genes involved in fatty acid synthesis by targeting downstream enzymes, promoting the production of fatty acid and cholesterol (53). ATF4 regulates adipogenesis by modulating PPARγ (peroxisome proliferator-activated receptor γ) through downstream CHOP. The differentiation of beige adipocytes is transiently regulated by decreased phosphorylation of eIF2α and CHOP, exacerbating the metabolic consequences of obesity by inhibiting adipogenesis and limiting lipid storage in adipose tissue (54). Therefore, strict control of eIF2α phosphorylation represents a pathway to optimize adipogenesis and ameliorate obesity and its metabolic dysfunctions. Furthermore, the extent and duration of eIF2α phosphorylation and its downstream integrated stress response can regulate the expression of PPARγ and C/EBP, which are key transcription factors for adipogenesis (55). These central regulators of adipogenesis interact positively to control and coordinate the expression of the entire adipogenic processes, including stimulating insulin-dependent glucose transport, inducing other transcription factors, and inhibiting growth-related genes (56). The impact of the ATF6 branch on adipose tissue function is relatively less studied. Research suggests that ATF6 can promote lipogenesis by activating SREBP-1, inducing the production of lipogenic markers such as fatty acid synthase(FAS), ACC2, and 3-hydroxy-3-methylglutaryl-coenzyme A (HMG-CoA) (57). *In vitro* studies have demonstrated that knockdown of ATF6 impairs adipogenesis and differentiation of preadipocyte cell lines into mature adipocytes (58). During glucose deprivation, ATF6 inhibits lipogenesis mediated by sterol regulatory element-binding protein 2 (SREBP2) (a substrate of S1P/S2P) (59). The overall process of how ERS regulates adipogenesis in the obese environment is illustrated in **Figure 2**. The response of the UPR to ERS controls the function of adipose tissue. On the other hand, dysfunction of adipose tissue can also influence changes in obesity-related inflammation, metabolic diseases, and ERS (60).

##### 2.2.2.2 Adipose tissue dysfunction affects ERS

Hypertrophy and hyperplasia of adipose cells are the basic features of adipose tissue in obesity. Chronic inflammation in obesity is closely related to inflammatory cell infiltration and cytokine network activation in adipose tissue (61). The phenotypic and biological changes of adipose tissue are also associated with metabolic diseases such as obesity, insulin resistance, inflammation and diabetes (62). Studies have found that ERS can promote obesity-induced insulin resistance, diabetes and hepatic steatosis, and can induce lipogenesis, which is a bridge between obesity and adipose tissue function (63). In the early stages of obesity, adaptive ERS/UPR response is activated to regulate the good function and metabolic homeostasis of adipose tissue. However, when excessive ERS exceeds the normal carrying capacity of obesity and fat cells, the maladaptive UPR triggers the cell toward the apoptotic pathway (64).

#### 2.2.3 Adipose tissue inflammation crosstalk ERS

##### 2.2.3.1 Adipose tissue macrophages

Adipose tissue is closely related to chronic inflammation in obesity to a large extent, and plays an important endocrine role in regulating immunity, inflammation and energy homeostasis (65). In the process of metabolic stress, the main way to promote adipose tissue inflammation is the crosstalk between immune cells and metabolic cells, that is, the interaction between macrophages and adipose cells, which plays an important role in the metabolic disorders caused by obesity (66). The role of the immune system in adipose tissue is one of the new hotspots in the study of metabolic regulation and obesity (67). Adipose tissue inflammation is closely related to the function of macrophages in adipose tissue, which are an important component of inflammation. There are two main types, M1 macrophages and M2 macrophages. The former tends to cause tissue damage and chronic inflammation (pro-inflammatory), while the latter tends to promote wound healing and eliminate inflammation (anti-inflammatory) (68). Obese adipose tissue macrophages (ATM) are M1-like macrophages, which are mainly involved in the chemokines secreted by adipose cells during lipid overload. At the same time, M1 macrophages can secrete a large number of chemokines and pro-inflammatory cytokines to attract more macrophages and amplify adipose tissue inflammation (69). Promoting the polarization of macrophages in obese adipose tissue is also a current therapeutic approach to treat obesity through the inflammatory pathway.

##### 2.2.3.2 Adipokines secreted by adipose tissue

Adipose tissue, as a crucial endocrine organ, produces adipokines, which are cell-signaling proteins that regulate various biological processes in target organs, including glucose and lipid metabolism, inflammation, endothelial cell function, angiogenesis, insulin sensitivity, and metabolic syndrome (70). In obese individuals, the balance between pro-inflammatory and anti-inflammatory adipokines is disrupted, leading to a gradual increase in pro-inflammatory adipokines within adipose tissue (71), which plays a critical role in the systemic homeostasis. This article briefly outlines the roles of the following adipokines in the cascade of inflammation and ERS in obese adipose tissue.

###### 2.2.3.2.1 Adiponectin

Adiponectin is a key adipokine involved in preventing adipose tissue inflammation and improving insulin sensitivity (72), primarily secreted by adipocytes in white adipose tissue. Adiponectin mainly reduces cellular lipid content through two pathways: one is by directly stimulating fatty acid oxidation and reducing fatty acid synthesis (73); the other is by regulating the circulating levels of insulin/insulin-like growth factor (IGF), promoting insulin/IGF sensitivity and action, thereby indirectly regulating fatty acid oxidation through the insulin/IGF system (74). Adiponectin activates PPARα, enhancing IRS signaling in the liver and skeletal muscle, while also increasing fatty acid oxidation, reducing intracellular lipid content, and improving insulin resistance (75). The production of adiponectin decreases with obesity, which is crucial for inflammation and also a significant factor in the development of obesity and atherosclerosis. Adaptor protein (APPL1) can transmit signals from adiponectin receptors to downstream targets, playing a crucial role in the cascade of adiponectin signaling. APPL1 serves as a key mediator in regulating fatty acid oxidation and glucose uptake, and is essential for adiponectin-induced activation of AMPK (adenosine monophosphate-activated protein kinase) and p38 mitogen-activated protein kinase (p38MAPK) activation (75). Research has demonstrated that inducers of ERS can lead to abnormal secretion of adipokines in adipose tissue (76). When the UPR cascade of ERS becomes dysregulated, IRE1 induces apoptosis in adipocytes through downstream JNK signaling (11). Further characteristics of this pathway include increased production of C/EBP homologous protein and decreased synthesis of adiponectin. Adiponectin plays a crucial role in regulating systemic fat deposition, insulin levels, and cellular lipid metabolism.

###### 2.2.3.2.2 Resistin

Resistin, a secretion factor exclusively present in adipose tissue, plays a pivotal role in insulin resistance associated with diabetes and obesity. In mice, resistin is primarily secreted by adipose tissue, whereas in humans, its main sources are cells other than adipocytes, including peripheral blood mononuclear cells, macrophages, and bone marrow cells (77). Although human resistin is primarily produced by macrophages rather than adipocytes, experimental studies indicate that human resistin exacerbates adipose tissue inflammation and leads to insulin resistance. Research has found that resistin can induce ERS, inhibit endothelial at serine residues, and consequently impair insulin phosphatidylinositol 3-kinase (PI3K)/serine/threonine kinase (Akt) signaling. Additionally, resistin signaling *in vivo* converges on common downstream targets, primarily activating NF-κB and MAPK (JNK/ERK1/2/p38) pathways. This modulation regulates pathways involved in upregulating the expression of pro-inflammatory genes (IL-6, TNF-α, monocyte chemoattractant protein-1), linking metabolic disorders such as insulin resistance, glucose homeostasis, and inflammatory response (78). There is also research indicating that ERS in adipocytes of obese mice can downregulate the expression of resistin in cultured mouse adipocytes, linking obesity and insulin resistance through adipose tissue (79–81).

Adipose tissue communicates with various organs by producing adipokines that impact organs such as the brain, heart, liver, and muscles. Released adipokines can exert effects on obesity, adipose tissue, adipocytes, and inflammation through the ERS response.

## 3 ERS-mediated inflammatory cascade

### 3.1 Overview of inflammation

Inflammation is a protective response in organisms, triggered by stimuli such as infection, chemicals, or physical injury, causing damage to host tissues or cells (82). Currently, inflammation is mainly classified into acute inflammation and chronic inflammation. The former is a transient response activated to eliminate stimuli and repair tissues, while the latter is a long-term response aimed ai eliminating pathogenic factors and/or repairing damaged tissues. Obesity, defined as abnormal or excessive accumulation of fat in adipose tissue, is considered a chronic inflammatory disease (83). The inflammation caused by obesity differs from inflammation in the general sense. In obesity, inflammatory triggers are metabolic, resulting from excessive consumption of nutrients (84). The inflammatory process in obesity primarily operates through the UPR pathway of ERS within the body. Evidence suggests that the initial process of inflammation is initiated by increased oxidative stress, triggered by impaired cellular functions such as ERS and mitochondrial dysfunction (85). Obesity, on the other hand, leads to excessive UPR, triggering inflammation and eliciting various cascading responses to inflammatory signals (86).

### 3.2 ERS mediates an inflammatory cascade through UPR

The crosstalk between the ER and inflammation primarily occurs through the UPR pathway. UPR aims to clear ER chaperones that persistently misfold and unfolded proteins accumulated within the ER to restore normal ERS (87). Simultaneously, the main purpose of UPR is to alleviate the burden of unfolded proteins, restore organelle homeostasis, reduce protein translation, and induce transcriptional components of the ER machinery. This is achieved through the induction of ER-associated degradation (ERAD) complexes, which promote the degradation of misfolded proteins (88). The main UPR reaction in inflammation are mediated by three branches: PERK, IRE1, and ATF6. UPR signals can directly interfere with inflammation-related pathways through various downstream branches, including JNK, IKK, and NF-κB signaling (89), as well as the production of reactive oxygen species (ROS) (90). Upstream studies identifying multiple inflammatory factor expressions have determined that JNK (91), IKK, and Akt are key intracellular factors inducing metabolic tissue inflammation (92). Akt can coordinate the activity of key inflammatory kinases (such as JNK and IKK), insulin receptor signaling components (such as IRS-1), and translation machinery (via eIF2α) in the UPR, tightly linking inflammation, metabolic dysregulation, and ERS functionality (12). The NF-κB/IKK pathway is a commonly used signaling pathway in inflammation, activated when cells are stimulated by various factors, including cytokines, growth factors, ROS, and microbial components such as lipopolysaccharide (LPS) (16). IKK is composed of two catalytic subunits (Ikkα and Ikkβ) and one regulatory subunit (Ikkγ). IKK is activated through the classical NF-κB signaling pathway. Ikkβ is one of the most important kinases mediating intracellular inflammatory stimuli (93). ERS can activate IKK through the classical NF-κB pathway, inducing the expression of downstream major regulatory factors (such as TNF-α) (94).

#### 3.2.1 PERK branch

Activation of PERK is primarily associated with the Ikk-NF-κB pathway. Upon ERS, PERK is activated, leading to phosphorylation of its downstream effector eIF2α (95). Phosphorylation of eIF2α and subsequent translational attenuation reduce the synthesis of IκB, followed by activation of the transcription factor NF-κB, as part of the stress response, thereby enhancing inflammation through NF-κB nuclear translocation (96). Research indicates that NF-κB can be activated through the inhibition of IκB translation via this pathway, leading to the regulation of inflammatory mediators such as IL-6 and TNF-α (97). TNF-α is a major pro-inflammatory cytokine that induces the transcriptional upregulation of pro-inflammatory molecules through the activation of MAPK and NF-κB pathways (98). TNF-α can also increase the release of free fatty acids (FFA) from adipocytes, inhibit the synthesis of adiponectin, and interfere with the phosphorylation activity of tyrosine residues on the insulin receptor substrate, thereby exerting insulin-resistance activity (99). Meanwhile, phosphorylation of downstream eIF2α by PERK increases the translation of downstream ATF4 and CHOP, enhancing the translation of stress-responsive genes that promote autophagy, thereby increasing the ability to maintain autophagy in stressed cells (100). ATF4 is a transcription factor that plays a crucial role in promoting survival, enhancing autophagy, ER folding capacity, antioxidant response, and amino acid metabolism, among other biological processes. ATF4 participates in cell apoptosis through the expression of CHOP protein (101), and it can induce eIF2α inhibition negative feedback release and glucose metabolism. CHOP translocates into the nucleus, where it can activate members of the BCL-2 (B-cell lymphoma 2) family such as Bcl-2-associated X protein (BAX), promoting inflammation and oxidative stress responses (102). Phosphorylation of eIF2α also activates nuclear factor erythroid 2-related factor 2 (NRF2) and PI3K. NRF2 directly interacts with NF-κB to induce inflammation. Under PI3K activation, downstream protein Akt is activated. Activation of Akt leads to the activation of mTOR (mammalian targe of rapamycin), which in turn participates in processes such as gene transcription, protein translation, ribosome biogenesis, integrating signals from extracellular nutrients, energy, and growth factors. These molecules are crucial for cell development, autophagy, and apoptotic metabolism.

#### 3.2.2 IRE1 branch

The IRE1 branch is considered the oldest and most conservative branch of the UPR, positioned at the intersection of several molecular pathways in response to cellular stress (103). Upon binding and dissociation from BiP, IRE1 undergoes dimerization, autophosphorylation, and activates ribonuclease (RNase) activity (104). IRE1 forms complex signaling platform with adaptor proteins on the ER membrane, exerting its effects by controlling the activation of the JNK and NF-κB pathways (105). Primarily through activated IRE1 recruiting tumor necrosis factor receptor (TNFR)-associated factor 2 (TRAF2) and apoptosis signal-regulating kinase 1 (ASK1), it mediates JNK and NF-κB activation (106). JNK is a crucial component of inflammatory signaling, capable of activating the activator protein-1 (AP-1) transcription factor complex, thereby increasing the expression of inflammatory factors such as IL-6 and TNF-α (107). Meanwhile, JNK can inhibit the downstream anti-apoptotic protein Bcl-2, promote BAX-dependent cell apoptosis, and coordinate cell death (108). The IRE1 branch mediates various inflammatory pathways through the IRE1-TRAF2 axis, affecting biological metabolism. As a core pathway, the IRE1-TRAF2 axis also activates the NF-κB inflammatory pathway by enhancing the interaction between nucleotide-binding oligomerization domain 1 and 2 (NOD1/2) receptors and serine/threonine protein kinase 2 (RIPK2) (109). TRAF2 plays a significant role in inflammation and biological metabolism through the UPR branch. On the other hand, TRAF2 directly interacts with IKK and promotes the degradation of IκB through IKK-mediated phosphorylation, thereby increasing the nuclear translocation of NF-κB, which is one of the important pathogenic mechanisms of obesity-related inflammation (110). Phosphorylation of JNK and IKK also impairs insulin action and glucose homeostasis (111).

#### 3.2.3 ATF6 branch

**Figure 3:**
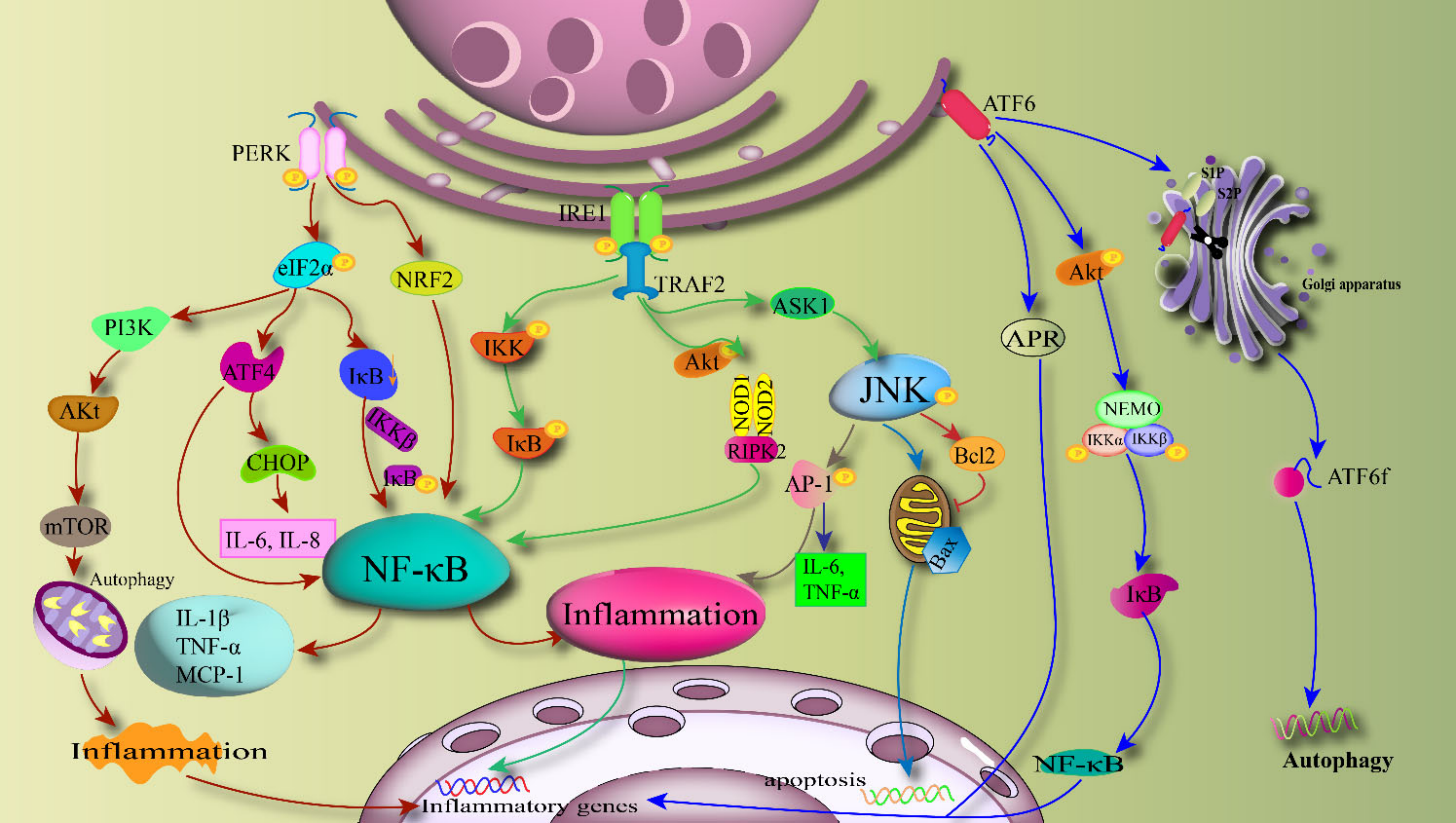
ERS-induced inflammatory cascade: PERK, IRE1 and ATF6 activate the inflammatory pathway with NF-κB, JNK and IKK as the core through the downstream branches, respectively. PERK directly activates NF-κB via NRF2 and also mediates Akt activation of autophagy and associated inflammatory transcription factors. IRE1 mainly mediates downstream inflammatory pathways through the IRE1-TRAF2 axis, and can also lead to apoptosis through Bcl2. ATF6 can induce autophagy through active forms and regulate inflammatory responses through the NF-κB pathway.

ATF6 is a member of the type II transmembrane receptor and leucine zipper protein family, with its N-terminal DNA-binding domain located in the cytoplasm and its C-terminal domain situated in the lumen of the ER (112). Under ERS stimulation, GRP78 dissociates from ATF6, and ATF6 is then transported to the Golgi apparatus through interaction with the protein transport vesicle coat protein COPII. ATF6 is cleaved by S1P and S2P in the Golgi apparatus into its active amino-terminal form (113). The active form can then enter the nucleus and regulate the expression of endoplasmic reticulum chaperone proteins (114). Similar to IRE1 and PERK, ATF6 also participates in inflammatory pathways by modulating NF-κB activity (115). ATF6 can induce the degradation of IκB by activating Akt and IKKα, β, γ. Under ERS, ATF6 can also influence the activation of acute phase reaction protein (APR) and activate the transcription of inflammatory genes in the cell nucleus. Processing of ATF6 generates active transcription factors that, in addition to targeting genes encoding endoplasmic reticulum chaperones, mediating inflammation, and ERAD components, also play crucial roles in lipid biosynthesis and endoplasmic reticulum expansion (116). It has been shown that ATF 6 can enhance the induction of ERAD proteins by forming heterodimers with spliced XBP1. The activation of ATF6 inhibits the induction of SREBP-mediated lipogenic genes by recruiting the corepressor histone deacetylase at the target gene promoter in hepatocytes, thereby regulating lipid metabolism (59). Additionally, research has demonstrated that ATF6 also exerts inhibitory effects on gluconeogenesis. It has been shown that ATF6 modulates the activity of the key transcription factor cAMP response element-binding protein (CREB) by competing with the transcriptional coactivator transducer of regulated CREB activity 2 (TORC2), thereby suppressing gluconeogenesis in the mouse liver (117). The increase in ERS promotes the expression of ER quality control genes through association with ATF6α. ATF6α reduces hepatic glucose output by disrupting the interaction between CREB and TORC2, thereby contributing to glucose homeostasis. ERS induces inflammatory cascades through the UPR pathway as illustrated **Figure 3**.

Obesity is a systemic chronic inflammation that plays a causative role in complications such as obesity-related insulin resistance and type 2 diabetes (118). Some well-known inflammatory pathways, such as protein kinases JNK, IKK, and NF-κB, are considered key molecular links between obesity, metabolic inflammation, and glucose homeostasis. Obesity is closely associated with inflammation pathways through the UPR pathway of ERS, with widespread activation of components and downstream signaling cascades (50).

## 4 Treatment of inflammation in obese adipose tissue

### 4.1 WATME-specific immunotherapy

Traditional methods of preventing or treating obesity typically include lifestyle changes, behavioral adjustments, dietary control, medication, or surgical interventions. However, obesity is regulated by a multitude of factors and variables, making it a highly complex disease. The conventional treatments have their limitations. Given the critical role of the immune system in regulating the metabolism, fat accumulation, and inflammation associated with obesity, a new therapeutic approach has emerged. This method focuses on alleviating inflammation in the white adipose tissue microenvironment (WATME) by targeting cells, signaling pathways, and secreted cytokines, known as “WATME-specific immunotherapy.” It plays a key role in treating obesity and obesity-induced diseases such as type 2 diabetes and coronary heart disease. The chronic systemic inflammation induced by obese WATME can lead to prolonged hyperinsulinemia, resulting in β-cell dysfunction and ultimately the development of type 2 diabetes. Additionally, the pro-inflammatory cytokines secreted by obese WATME are closely linked to the development of atherosclerosis. Therefore, employing WATME-specific immunotherapy to inhibit the secretion of pro-inflammatory cytokines can effectively treat diseases induced by obesity, such as type 2 diabetes and coronary heart disease (119).

### 4.2 Nanoparticle therapy

The inflammatory microenvironment is considered a potential therapeutic target for treating metabolic diseases associated with obesity, and modulating the adipose tissue microenvironment is highly promising. Studies have tested (120) the treatment of obesity through nanoparticles that induce increased energy expenditure and regulate the adipose tissue microenvironment, creating an inflammatory environment within adipose tissues. Various nanomodulators have been applied locally in WAT, and the results show that under nanoparticle treatment, the levels of TNF-α and the number of macrophages significantly decrease. The findings prove that nanoparticles loaded with Amlexanox and modified with anti-VCAM-1 antibodies can increase energy expenditure, prevent the development of obesity, and simultaneously, the particle system can remodel the adipose tissue microenvironment, improving inflammation in adipose tissues and alleviating systemic metabolic disorders.

### 4.3 Targeting ERS for treatment

ERS and its downstream UPR branches play pivotal roles in linking inflammation with the microenvironment of obese adipose tissue. Approaches to regulate ERS and thereby affect inflammation in adipose tissues are gaining attention. Recent studies (121) have uncovered that dysregulation of miRNAs can impact the functionality of fat, liver, and muscle tissues. Knockout studies have shown that miR-149 may influence ERS through negative targeting of the ATF6 signaling pathway. Additionally, research suggests that miRNAs could represent a potential mechanism for regulating the renin-angiotensin system (RAS) signaling pathways. Treating adipocytes with angiotensin II alters miRNAs targeting ERS and inflammation, leading to adipocyte dysfunction.

Regarding the XBP1 branch, studies employing XBP1 inhibitors to intervene in obesity have demonstrated the ability to alleviate abnormal ERS and oxidative stress within fat cells, thereby significantly inhibiting abnormal fat formation, reducing lipid droplet accumulation, and blocking triglyceride synthesis to prevent cascading adipocyte proliferation and the progression of obesity (122). Furthermore, it has been discovered that ubiquitination of proteins associated with endoplasmic reticulum stress can exacerbate the intercellular transmission of ERS signals, resulting in adipocyte dysfunction and insulin resistance (123).

Therefore, targeting ERS represents a potential therapeutic target for treating inflammation in obese adipose tissue and obesity-related complications. This strategy highlights the importance of understanding the molecular pathways involved in ERS and its connection to obesity and its sequelae, opening avenues for developing targeted interventions to combat obesity and its associated disorders.

## 5 Conclusion

Obesity is a major risk factor for hypertension, arteriosclerosis, type 2 diabetes, insulin resistance, ischemic heart disease, dyslipidemia, and other metabolic disorders. The prevalence of obesity is increasing globally, yet pharmacological treatments for obesity and its associated diseases are significantly limited. Understanding the pathogenesis of obesity is crucial for its treatment. This article reviews the mechanisms by which endoplasmic reticulum stress affects obesity, adipose tissue, and related inflammation. It highlights the role of the endoplasmic reticulum in interacting with insulin signaling, inflammatory signals, carbohydrate and lipid metabolism, cell proliferation, autophagy, and apoptosis through UPR branches, playing a significant role in metabolic diseases characterized primarily by metabolic damage such as inflammation, nutritional metabolic damage, and insulin resistance. Adipose tissue, as an important endocrine organ in obesity, targets the treatment of obesity through the interaction of adipose tissue endoplasmic reticulum stress, secreted adipokines, and inflammation.

Furthermore, ERS is inextricably linked to the onset and progression of various metabolic diseases associated with obesity. The UPR branches of ERS tightly connect obesity, inflammation, and insulin resistance. Nitrosylation of its branch IRE1 can impair the UPR, thereby promoting insulin resistance in obese mice. Targeting the kinase activity of IRE1 may be beneficial for metabolic syndrome and inflammatory lipid disorders (124). Thus, targeting ERS to inhibit the secretion of pro-inflammatory cytokines can effectively treat complications such as type 2 diabetes and coronary heart disease. Additionally, the UPR, as a cellular response activated in ERS, also plays a key role in the pathogenesis of non-alcoholic fatty liver disease (NAFLD), hepatitis, and hepatocellular carcinoma. In NAFLD, lipid accumulation triggers liver ERS and activates the UPR response. Phosphorylated IRE1α can induce the expression of XBP1s and caspase-2, leading to liver steatosis, hepatocyte damage, and insulin resistance (IR). Similarly, phosphorylated PERK induces the phosphorylation of eIF2α and the expression of ATF4, further promoting the pathological process of liver steatosis (125). Currently, the treatment for NAFLD is based on dietary control, physical activity, and surgical weight loss, but the UPR has recently been proven to be an ideal target for various drugs aimed at alleviating the progression of NAFLD. This underscores the significant research value of studying ERS and its UPR branches’ mechanisms (126, 127).

Research on weight loss medications has always been a hot topic. Currently, systemically administered anti-obesity drugs approved by the FDA primarily act by manipulating central nervous system pathways or by inhibiting appetite and reducing fat absorption through the gastrointestinal tract. However, these methods may trigger stimulant or depressant-like syndromes related to the nervous system and potentially increase the metabolic load on the kidneys. Thus, there is an urgent need to explore new strategies for treating obesity and its complications that can enhance pharmacological safety. Many basic studies targeting ERS have identified potential targets for treating obesity and its complications. However, clinical studies based on ERS targets are relatively scarce, and the development of clinical drugs still faces certain challenges. Medications and clinical trials capable of targeting ERS to effectively reduce weight and alleviate obesity complications are eagerly awaited for further research. This review analyzes the mechanisms of action in targeting ERS for the treatment of inflammation in obese adipose tissue, with the hope that subsequent research will focus more on ERS as a target, providing potential targets for the treatment of obesity and its metabolic diseases.

## Author contributions

KM: Writing – original draft, Writing – review & editing. YZ: Conceptualization, Writing – review & editing. JZ: Conceptualization, Writing – review & editing. LZ: Writing – review & editing. ML: Supervision, Writing – original draft, Writing – review & editing.
